# Effect of crude protein content and undegraded intake protein level on productivity, blood metabolites, carcass characteristics, and production economics of Hanwoo steers

**DOI:** 10.5713/ajas.19.0822

**Published:** 2020-02-25

**Authors:** Youn Hee Lee, Farhad Ahmadi, Myun Lee, Young-Kyoon Oh, Wan Sup Kwak

**Affiliations:** 1 Department of Food Bio-science, College of Medical Life Sciences, Konkuk University, Chungju 27478, Korea; 2 Animal Nutrition & Physiology Team, National Institute of Animal Science, Rural Development Administration, Wanju 55365, Korea

**Keywords:** Carcass Quality, Crude Protein, Hanwoo, Marbling, Undegraded Intake Protein

## Abstract

**Objective:**

This study was designed to determine how feeding diets differing in crude protein (CP) and undegraded intake protein (UIP) levels affected productivity, blood metabolites, carcass characteristics, and the production economics of Hanwoo steers.

**Methods:**

Thirty-six Hanwoo steers (age = 8.2±0.5 mo; body weight = 254±16.1 kg) were assigned at random to one of three treatments (4 steers/pen; 3 pens/treatment): i) a low-CP diet (LP; control) containing 12.1% CP with 35.1% UIP, 12.0% CP with 36.8% UIP, and 12.9% CP with 48.8% UIP, in the growing, fattening, and finishing periods, respectively; ii) a high-CP, low-UIP diet (HPLU) containing 15.0% CP with 33.7% UIP, 14.0% CP with 35.7% UIP, and 13.1% CP with 46.7% UIP, respectively; and iii) a high-CP, high-UIP diet (HPHU) containing 15.0% CP with 45.8% UIP, 14.0% CP with 44.6% UIP, and 13.0% CP with 51.1% UIP, respectively.

**Results:**

The treatments did not affect feed intake and growth performance, except for average daily gain during the fattening period that tended to be the lowest (p = 0.08) in the HPLU-fed steers. The feed CP conversion ratio over the entire feeding period was higher with high-CP diets. The treatments did not affect most blood metabolites; however, blood cholesterol and low-density lipoprotein concentrations during the fattening and finishing periods were the lowest in steers fed a HPLU diet. The treatments had negligible effects on cold carcass weight, yield traits including *longissimus* muscle area, backfat thickness, yield index, and yield grade, plus quality traits including meat color, fat color, texture, and maturity. However, marbling score and frequency of carcass quality grade 1^++^ were greater in HPHU-fed steers.

**Conclusion:**

Feeding diets with higher CP and UIP levels did not affect growth performance but tended to improve the carcass quality of Hanwoo steers, resulting in greater economic return.

## INTRODUCTION

Huuskonen et al [1] found in their meta-analysis study that increasing crude protein (CP) concentrations in growing cattle diets were associated with increased weight gain and feed conversion ratio. However, this positive response declined with increasing mean body weight (BW) of animals. Additionally, management factors that decrease dry matter intake (DMI) as well as day-to-day variations in milling and delivery necessitate formulating diets with higher levels of CP than suggested by standard guidelines, which may provide a safety factor to offset these variables. Based on anecdotal evidence, some formulators of total mixed ration (TMR) for Hanwoo cattle in Korea usually use higher concentrations of CP than suggested by the Korean Feeding Standard Establishment Council [2] and National Research Council [3], regardless of ruminal degradability, to ensure the required CP content is provided and to maximize cattle performance.

Although increasing CP concentrations usually improves the performance in growing/finishing beef cattle, animal responses to increased CP content vary with respect to the CP source (i.e. ruminal degradability level) [4]. This is the reason that most recent CP systems for beef cattle take into account both quantity and quality of dietary protein. Generally, degraded intake protein (DIP) is required to facilitate microbial protein synthesis in the rumen. Nutritionally, it is important to maximize the absorbed amino acids in the small intestine coming from ruminal microbial protein and undegraded intake protein (UIP), which necessitates the proper level of UIP and DIP in the diet to maximize animal performance [5]. Compared to mature animals, growing cattle require increased quantity and quality of dietary protein to meet their elevated demand for amino acids required for muscle accretion.

The effects of manipulating protein levels as well as the ratio of DIP to UIP on growth performance and carcass quality parameters of beef cattle are contradictory in literature. For example, Vasconcelos et al [6] reported that increased dietary protein concentrations increased marbling development in crossbred steers. Faucitano et al [7] also reported greater marbling scores in steers fed diets with higher UIP concentrations. However, Gleghorn et al [8] and Koenig et al [9] found no differences in the effects of varied CP concentrations or the UIP:DIP ratio on marbling development in beef cattle. These inconsistencies justify more extensive investigations to elucidate the effects of CP and UIP levels on performance and meat quality parameters in beef cattle, especially in native breeds other than Holstein cattle such as Hanwoo (Korean native cattle), which has received little attention. We hypothesized that an increased supply of CP and UIP might improve the productivity and carcass traits of Hanwoo steers. Therefore, the aim of this study was to determine the appropriate levels of CP and UIP in Hanwoo steer diet over the growing, fattening, and finishing periods, and to investigate the effects on productivity, selected blood metabolites, carcass characteristics, and production economics of Hanwoo steers when slaughtered at an average age of 30 mo.

## MATERIALS AND METHODS

### Animals, treatments, and management

All procedures related to the animals were pre-approved by the Institutional Animal Care and Use Committee of the Konkuk University. Thirty-six Hanwoo steers were purchased from a commercial livestock order-buyer market and transported to the Animal Experimental Farm of Konkuk University (36° 58′ N latitude and 127° 57′ E longitude and an elevation of 49 m above sea level). After being acclimated to farm conditions, steers with an average age of 8.2±0.5 mo and weight of 254±16.1 kg, were assigned at random to one of three diets differing in CP and UIP content (4 steers per pen; 3 pens per treatment): i) a low-CP diet (LP; control) containing 12.1% CP with 35.1% UIP, 12.0% CP with 36.8% UIP, and 12.9% CP with 48.8% UIP, in the growing, fattening, and finishing periods, respectively; ii) a high-CP, low-UIP diet (HPLU) containing 15.0% CP with 33.7% UIP, 14.0% CP with 35.7% UIP, and 13.1% CP with 46.7% UIP, respectively; and iii) a high-CP, high UIP diet (HPHU) containing 15.0% CP with 45.8% UIP, 14.0% CP with 44.6% UIP, and 13.0% CP with 51.1% UIP, respectively. To minimize the effects of dietary factors on animal performance, diets were formulated to provide similar concentrations of energy and nutrients. Diets differed only in the level of CP or the ratio of UIP to DIP supplied. UIP and DIP balances were adjusted primarily by changing the dietary proportion of soybean meal (SBM) as a CP source with high DIP, and a high-protein corn dried distillers grain (DDG) as a CP source with high UIP. The UIP content of the SBM and the high-protein DDG was determined in our previous *in situ* study [10] as 15.0% and 61.0% of CP, respectively. The UIP content of other feed ingredients was obtained from standard values specified by the Korean Feeding Standard Establishment Council [2]. The chemical compositions of the individual feed ingredients are provided in Table 1. The chemical compositions of experimental diets are presented in Table 2.

Diets were offered as TMR twice a day at 07:00 and 18:00 h. The entire experiment lasted 21.1 mo. The growing phase was from 8.2 to 14.3 mo. The fattening phase started at an age of 14.3 mo and ended at 23.5 mo. The finishing period started at an age of 23.5 mo and the steers were slaughtered at 29.3 mo. Fresh water was accessible during the entire feeding period. Feed samples were obtained weekly, frozen, and composited within each mo of the experiment. The BW measurements were read using a digital weighing indicator (Model: CI-200A Series, CAS Corporation, Seoul, Korea). Feed intake was measured on a pen basis. The feed conversion ratio was calculated as daily DMI or CP intake per average daily gain (ADG).

### Sampling and laboratory analyses

Dry matter, CP, ether extract, crude ash, neutral detergent fiber, and acid detergent fiber were determined according to standard procedures specified by the Association of Official Analytical Chemists [11]. At ages of 13.6, 22.5, and 27.5 mo, blood samples were collected 4 h after the morning feeding via jugular venipuncture into vacutainers without (BD Vacutainer SST II Advance; Plymouth, UK) or with an anticoagulant (BD Vacutainer, spray-coated K2EDTA, Franklin Lakes, NJ, USA). Blood hematological parameters were determined in the vacutainers with anticoagulant. Serum was harvested by centrifugation (2,000×g, 15 min), and stored at −20°C until analysis. The vacutainers were sent to a commercial laboratory (Green Cross Corporation, Yongin, Korea) for quantification of metabolites. Calcium, phosphorus, potassium, sodium, and chlorine concentrations were quantified in blood samples using an inductively coupled argon plasma emission spectroscopy (ICP-OES 5300DV, Perkin Elmer, Billerica, MA, USA).

### Carcass evaluation

At an age of 24 mo, the live-animal carcass measurements were made by trained technicians using an ultrasound machine (Aloka SSD-500, Hitachi Aloka Medical, Ltd., Wallingford, CT, USA). The measurements were made on the left side of the animal between the 12th and 13th ribs. For evaluation of after-slaughter carcasses, 24 hours prior to slaughter, diets were withdrawn from the steers. Following a 48-h post-mortem chill, each carcass was evaluated for yield and quality grade by official graders using the Korean Carcass Grading Standards [12].

### Production economics

An economic analysis of the total feed cost for each period was based on DMI, days on feed, and TMR cost per kg. Gross income was calculated as the selling price of the graded carcass and associated by-products. The operating costs were calculated as the sum of the average purchased price of steers at the start of experiment and total feed costs. Net income was calculated as the difference between total gross income and operating costs.

### Data analysis

Data were analyzed using the Proc Mixed of SAS (SAS Institute, Cary, NC, USA, 2003) with steer or pen being the experimental unit, depending on the response variable. For statistical analysis of nutrient intake data, pen served as experimental unit. Dietary treatments were considered as a fixed effect and steer or pen as a random effect. Initial BW was included in the model as a covariate. To properly account for within-steer correlations for repeatedly-measured data, the first-order autoregressive [AR(1)] was included in the model. When a significant effect was detected at a p-value less than 0.05, comparisons between means were made using the Tukey’s multiple range test.

## RESULTS

### Performance

No steer died or was removed from the experiment and over the entire experimental period all steers remained healthy without displaying any sign of illness. Treatment effects on the performance of Hanwoo steers according to the production phases are presented in Table 3. At the end of the growing period, final BW did not differ among treatments (401±6.0 kg). Steers started the finishing phase with an initial BW of 599±22 kg and were harvested at the average BW of 742±25.1 kg. The treatments also had no effects on ADG during the growing and finishing phases. However, ADG tended to be the lowest (p = 0.08) in steers fed the HPLU diet during the fattening period. The treatments did not affect DMI during the growing, fattening, and finishing phases. As expected, because there were no differences in DMI among treatments during the growing and fattening periods CP intake increased (p<0.01) as dietary CP level increased. However, during the finishing period, CP intake was not different across diets. Feed conversion ratios during the entire feeding period, expressed as DMI divided by ADG, were not affected by CP concentration or degradability. However, feed conversion ratios, defined as CP intake divided by ADG, over the entire feeding period were higher with high-CP diets.

### Blood chemistry and hematology

Treatment effects on selected blood metabolites during the different production phases are presented in Table 4 and 5. No differences existed in triglyceride, high-density lipoprotein, glucose, and total protein concentrations among treatments. This possibly suggests that treatments had negligible effects on steer fat, energy, and protein metabolism. Cholesterol and low-density lipoprotein concentrations collected at an age of 13.6 mo were not different across treatments; however, concentrations of these metabolites at 22.5 and 27.5 mo were lower in steers fed high-CP diets. Although treatments had no effects on blood urea-N (BUN) concentrations harvested at ages 13.6 and 27.7 mo, HPLU-fed steers had the greatest BUN concentrations (p = 0.03) at 22.5 mo. Treatments had no effects on the blood activity of circulating enzymes originating from the liver, alkaline phosphatase, and lactate dehydrogenase. Blood electrolytes were not affected by treatments at any sampling time.

### Carcass quality parameters

Ultrasonic measurements showed that concentration or ruminal degradability of CP had no effects on backfat thickness and the *longissimus* muscle (LM) area (Table 6). However, marbling scores tended to be greater (p = 0.08) in the HPHU-fed steers. A numerical improvement (p = 0.14) was seen in the carcass quality grade of steers fed the HPHU diet; for example, 25% and 75% of the carcasses qualified as 1^+^ and 1 grades, respectively.

Treatment effects on carcass characteristics after slaughter are presented in Table 7. Marbling scores tended (p = 0.05) to be highest with HPHU-fed steers. Overall, steers receiving the high-CP diet with less ruminal degradability (HPHU) tended to have the better carcass quality grades. For example, the proportion of carcasses graded ≥1^+^ were greatest with steers fed HPHU (83.3%), intermediate with LP (50.0%), and lowest with HPLU (41.6%). Treatments did not affect cold carcass weight, yield traits (including backfat thickness, yield index, and yield grade), and quality traits (including meat color, fat color, texture, and maturity). Although non-significant, LM area increased an average of 6 cm^2^ for high CP-fed groups when compared to the control group. However, this numerical difference might be of practical importance in the beef cattle market.

### Production economics

Average feed costs calculated for each experimental diet according to the growing, fattening, and finishing periods, as well as the production economics of Hanwoo steers, are presented in Table 8. Average total feed costs (from ages 8.2 to 29.3 mo) were 3.7% and 2.4% greater for HPLU and HPHU than that for the control group, respectively. However, this difference was negligible across treatments. Net income was the greatest for steers fed HPHU ($2,844), followed by LP-fed steers ($2,646), and HPLU-fed steers ($2,365). Therefore, in comparison with HPLU and LP, the HPHU diet resulted in 20% and 8% higher net income per steer, respectively.

## DISCUSSION

### Productivity

Typically, the initial phase of growth period is when feed intake is relatively low and the rate of protein deposition is potentially high. Therefore, increasing CP concentration during this phase may increase the growth rate. However, as the animal matures, with the concurrent increase in DMI and a proportionally lower rate of protein deposition, an excessive supply of protein to the animal may negatively affect its growth performance [6,8]. Therefore, the CP level of diets slightly decreases as the growing period ends and the fattening and finishing phases of steers are initiated (Table 2).

The slight difference in growth performance agrees with a previous report on beef cattle, where a dietary CP reduction from 14.5% to 10.8% had a marginal effect on growth performance [13]. Likewise, Dal Maso et al [14] reported that growth performance was negligibly affected when dietary CP concentrations decreased from 14.7% to 11.0% in double-muscled crossbred young bulls. Conversely, Jeong et al [15] studied the responses to low- (12%) and high-CP (14%) diets with a similar total digestible nutrient (TDN) content (75%) in the finishing Hanwoo steers, and found that the high-CP diet increased final BW and feed conversion ratio while slightly affecting DMI.

Generally, the shortage of N and an imbalance in the UIP:DIP ratio for ruminal microorganisms are associated with a reduction in total-tract nutrient digestibility, with subsequent depressions of DMI and growth performance [16,17]. Basically, the high-UIP diets improve N retention, N utilization, and metabolizable protein supply in growing beef cattle, thereby increasing their growth efficiency [18]. However, the oversupply of UIP may place an increased demand to ammonia detoxification and N recycling, which in turn may increase energy expenditures and compromise animal performance [19]. Contrary to these reports, in the current experiment the differences in CP and degradability levels of diets slightly affected DMI and growth performance (Table 3).

### Blood metabolites

Concentrations of all blood metabolites fall within normal ranges previously reported for Hanwoo steers [20-22]. Schiavon et al [16] examined effects of diets differing in CP level (14.5% vs 10.8%) in young Piemontese bulls, and reported that blood concentrations of total protein, creatinine, γ-glutamyl transferase, aspartate aminotransferase, triacylglycerol, and glucose were not affected by dietary treatments. The observation that blood levels of total protein and albumin were not affected by treatment was expected because their concentrations are typically reflected in BW change [21], which differed marginally across diets (Table 3).

A negative correlation has been reported between protein intake and serum cholesterol concentration in ruminants [23]. Although the mechanism underlying the cholesterol-depressing effect of dietary protein is poorly understood, dietary protein has been suggested to play an important role in the metabolism of the lipoprotein-lecithin:cholesterol acyltransferase complex. This complex is known to control the cholesterol-lipid distribution in the liver [23]. Cholesterol is the chief component of lipoproteins, which may explain why the serum concentration of low-density lipoprotein was lowest in steers fed the HPLU diet. A growing body of evidence confirms the hypocholesterolemic effect of soybean protein in several animal species. Although the exact mechanism is poorly understood, it is believed that feeding soybean protein impairs cholesterol absorption and/or bile acid re-absorption [24]. Moreover, many chemicals in soybeans, such as lecithin, saponins, fiber, phytic acid, and the isoflavones, are known to play a role in the hypocholesterolemic effects of soybean [24]. In the current experiment, HPLU-fed steers had the lowest serum concentration of cholesterol, which might be explained by the presence of SBM in this dietary treatment.

Feeding excessive amounts of DIP is associated with higher BUN concentrations, which is possibly caused by deamination of amino acids [25]. Ruminants fed CP sources with a high ruminal degradability are expected to have greater BUN concentrations than those fed supplemental N sources with less ruminal degradability [17]. Therefore, the higher BUN concentration in HPLU-fed steers during the fattening period was expected as this diet had the highest amount of DIP. It is plausible that the fast degradation of CP in the HPLU diet might have exceeded the capacity of ruminal microorganisms to efficiently utilize the ruminal ammonia nitrogen. Therefore, the excess ammonia nitrogen might have escaped from the rumen, thus resulting in an increased serum BUN concentration [26]. The HPHU diet did not result in an elevated concentration of BUN, which probably indicates the efficient use of protein fractions in the HPHU diet. However, in a study with Hanwoo steers, BUN concentrations increased as CP levels increased, irrespective of ruminal CP degradability [27].

### Carcass characteristics

Past studies with Hanwoo steers indicated that marbling scores reached an asymptotic level when cattle weighed about 570 kg, with an extended feeding beyond this weight only slightly benefiting carcass quality [28,29]. In the present experiment, the ultrasonic-carcass measurements were performed at the end of the fattening period, when the BW of steers averaged 600 kg. However, when the experiment continued to an approximate age of 30 mo, the meat quality grade improved. For example, at 24 mo (at ultrasound measurement), none of the carcasses qualified as 1^++^ grade; however, at harvest the likelihood of an individual carcass qualifying as 1^++^ (the highest quality) increased. Vasconcelos et al [6] also found that steers fed a diet with 13.0% CP had greater marbling scores than those fed with lower CP diets (11.5% or 10.0%); however, backfat thickness, LM area, and yield grade differed negligibly by CP concentration. In a study with finishing Hanwoo steers, Jeong et al [15] compared the effects of diets differing in CP concentration (12% vs 14%) and energy density (73% vs 75% TDN) and found that feeding with higher CP and TDN concentrations increased LM area, marbling score, meat quality grade, and improved production economics. In the present study, marbling scores tended to be numerically greater in the HPHU diet, which highlights the importance of UIP concentration, regardless of CP concentration, in marbling development. Faucitano et al [7] reported the higher UIP diets increased amino acids in the duodenum, which tended to increase the marbling score in the *longissimus dorsi* muscle of steers. The authors associated this phenomenon to the increased availability of specific amino acids used for gluconeogenesis in the liver, which then increased the availability of glucose in the muscle. Contrary to the findings of this study that HPHU diet promoted marbling development, Wagner et al [19] found that dietary UIP levels had no effects on carcass characteristics of heavy yearling steers. However, for the high-CP diet (14.5%), as UIP percentage increased, the marbling scores tended to decline. Gleghorn et al [8] and Koenig et al [9] also found that the difference in CP concentrations or degradability had minor effects on marbling scores and carcass characteristics. These discrepancies might be explained by the unique beef cattle feeding program in Korea that requires a long feeding duration until slaughter (usually 28 to 30 mo). Previous studies have suggested that as carcass weight increases, backfat thickness and LM area also increase in Hanwoo cattle [28,29], which may provide an explanation as to why the treatments did not affect LM area and backfat thickness in the current study, since carcass weights were not different across treatments (Table 7). Perkins et al [30] clarified that BW explains a large part of the variation in the LM area, which may provide evidence for the lack of differences in the LM area among treatments.

### Production economics

Marbling is the principal determinant of meat quality and price by meat distributors and consumers in the Korean beef cattle market, as increased marbling is associated with improved eating quality traits such as tenderness, flavor, and juiciness [29]. This indicates that producing well-marbled beef meat would greatly contribute to the profitability of beef cattle production [29]. In the current experiment, a greater economic return from steers receiving the HPHU diet was primarily due to the higher quality grade of the carcasses. Therefore, feeding high-CP diets with higher UIP levels is preferred from a cost of carcass by grade perspective for maximizing profit.

## CONCLUSION

Overall, the findings of this experiment indicate that despite of no meaningful effects on productivity, feeding a diet with higher CP and UIP levels improved the carcass quality of Hanwoo steers, which thus resulted in greater economic return.

## Figures and Tables

**Table 1:** Chemical composition of individual feed ingredients used in the experimental diets

Ingredients	Chemical composition (% of DM unless stated)										

	DM (%)	CP	EE	Ash	NDF	ADF	NFC	TDN1)	TP (% of CP)	DIP (% of CP)2)	UIP (% of CP)2)
Rice straw	81.9	4.03	0.81	11.2	71.7	46.1	12.3	53.3	61.6	80.0	20.0
Tall fescue hay	90.0	5.01	0.92	5.30	71.1	43.1	17.7	50.0	66.6	65.0	35.0
Soybean curd residue	26.8	22.4	5.01	3.73	33.0	26.0	35.9	81.4	69.2	50.0	50.0
Corn grain, cracked	89.1	8.12	3.10	2.34	14.6	5.56	71.8	86.3	99.1	35.0	65.0
Corn grain, flaked	88.8	7.98	3.41	2.02	13.9	6.11	72.7	89.1	95.3	35.0	65.0
Spent mushroom substrate	32.1	12.2	0.84	5.20	68.4	55.6	13.4	61.9	50.1	50.0	50.0
Wet brewers grain	31.3	19.9	6.20	4.51	70.0	34.2	−0.61	70.0	74.2	47.0	53.0
Corn gluten feed	93.7	21.2	5.21	9.44	44.6	13.8	19.6	73.3	38.2	75.0	25.0
Citrus pulp	18.1	8.01	1.43	4.81	28.4	27.6	57.4	74.7	47.3	65.0	35.0
Soybean meal	89.1	50.6	1.45	7.41	34.5	15.9	6.04	76.6	89.9	85.0	15.0
Lupin seed	86.9	33.6	5.10	3.45	31.5	26.9	26.4	80.3	79.2	67.0	33.0
High-protein corn DDG	89.6	38.8	1.66	2.21	54.3	23.4	3.03	83.6	93.0	39.0	61.0
Rice bran	91.2	13.5	18.5	10.0	27.8	18.6	30.2	88.3	-	50.0	50.0
Cottonseed, whole	90.3	20.0	10.6	3.90	47.0	42.3	18.5	80.9	81.2	55.0	45.0
Sugarcane molasses	72.4	5.42	0.10	12.7	7.61	0.60	74.2	85.0	64.6	90.0	10.0
Limestone	99.2	0.23	0.11	99.7	-	-	-	-	-	-	-
Urea	94.5	274	0.73	0.62	-	-	-	-	-	100	0
NaCl	88.8	0.31	0.10	89.2	-	-	-	-	-	-	-
Mineral-vitamin premix3)	92.8	10.5	4.32	46.5	-	-	-	-	-	-	-

DM, dry matter; CP, crude protein; EE, ether extract; NDF, neutral detergent fiber; ADF, acid detergent fiber; NFC, non-fibrous carbohydrates; TDN, total digestible nutrients; TP, true protein; DIP, degraded intake protein; UIP, undegraded intake protein; DDG, dried distillers grains.

1) TDN values were obtained from the mean values presented in the Korean Feeding Standard Establishment Council [2].

2) UIP and DIP contents of soybean meal and high-protein DDG were determined in our *in situ* experiment [10]. For other feed ingredients, DIP and UIP values were obtained from the mean values presented in the Korean Feeding Standard Establishment Council [2].

3) Provided (per kg): 107 mg of FeSO_4_, 57 mg of ZnSO_4_, 43 mg of MnSO_4_, 23 mg of CuSO_4_, 0.5 mg of CaI, 0.2 mg of CoSO_4_, 0.2 mg of Se, 3,000,000 IU of vitamin A, 330,000 IU of vitamin D_3_, 33,000 IU of vitamin E. Each value is the mean of 4 replicates.

**Table 2:** Ingredients and chemical composition (% DM, unless otherwise noted) of diets fed to Hanwoo steers during the growing, fattening, and finishing periods

Ingredients	Treatment								

	Growing			Fattening			Finishing		
		
	LP	HPLU	HPHU	LP	HPLU	HPHU	LP	HPLU	HPHU
Rice straw	15.0	15.0	14.0	14.0	14.0	14.0	10.1	10.1	9.0
Tall fescue hay	14.9	14.9	14.9	10.5	10.5	10.5	-	-	-
Soybean curd residue	14.0	14.0	13.4	14.0	14.0	14.0	16.0	16.0	16.0
Corn grain, cracked	12.3	10.5	12.8	21.3	17.3	16.7	-	-	-
Corn grain, flaked	-	-	-	-	-	-	43.9	41.9	42.0
Spent mushroom substrate	12.1	12.1	12.1	7.0	7.0	4.9	-	-	-
Wet brewers grain	11.2	11.2	11.2	8.5	8.5	9.0	13.0	13.0	13.0
Corn gluten feed	7.9	5.9	-	6.5	6.0	6.0	3.2	3.2	3.2
Soybean meal	-	5.8	-	-	3.5	-	-	2.0	-
High-protein corn DDG	-	-	14.0	-	-	5.70	-	-	3.0
Lupin seed	-	-	-	-	-	-	2.0	2.0	2.0
Rice bran	7.0	5.0	2.0	5.0	6.0	6.0	-	-	-
Citrus pulp	-	-	-	6.5	6.5	6.5	4.0	4.0	4.0
Cottonseed, whole	2.0	2.0	2.0	3.0	3.0	3.0	3.0	3.0	3.0
Sugarcane molasses	1.9	1.9	1.9	1.9	1.9	1.9	3.3	3.3	3.3
Limestone	0.7	0.7	0.7	0.8	0.8	0.8	0.7	0.7	0.7
Urea	0.4	0.4	0.4	0.4	0.4	0.4	0.2	0.2	0.2
NaCl	0.2	0.2	0.2	0.2	0.2	0.2	0.2	0.2	0.2
Mineral-vitamin premix1)	0.1	0.1	0.1	0.1	0.1	0.1	0.1	0.1	0.1
Microbial culture2)	0.3	0.3	0.3	0.3	0.3	0.3	0.3	0.3	0.3
Chemical composition3)									
DM (%)	64.1	64.8	65.1	64.0	63.2	63.0	64.1	64.2	64.4
CP	12.1	15.0	15.0	12.0	14.0	14.0	12.9	13.1	13.0
DIP (% of CP)	64.9	66.3	54.2	63.2	64.3	55.4	51.2	53.3	48.9
UIP (% of CP)	35.1	33.7	45.8	36.8	35.7	44.6	48.8	46.7	51.1
DIP:UIP	1.85	1.97	1.18	1.72	1.80	1.24	1.05	1.14	0.96
Ether extract	3.60	3.30	3.30	4.01	4.12	4.61	3.80	3.70	3.90
Ash	8.40	8.50	8.10	8.51	9.03	8.82	6.00	6.20	5.80
NDF	56.5	56.9	55.4	43.5	43.6	43.8	25.8	25.9	26.1
ADF	37.9	36.0	35.7	26.0	26.2	26.2	14.8	14.9	15.1
NFC	19.4	16.3	18.2	32.0	29.3	28.8	51.5	51.1	51.2
TDN	63.2	63.1	63.1	68.9	68.9	68.9	79.4	79.3	79.3

DM, dry matter; LP, low-protein diet; HPLU, high-protein, low UIP diet; HPHU, high-protein, high UIP diet; DDG, dried distillers grains; CP, crude protein; DIP, degraded intake protein; UIP, undegraded intake protein; NDF, neutral detergent fiber; ADF, acid detergent fiber; NFC, non-fibrous carbohydrates; TDN, total digestible nutrients.

1) Provided (per kg): 107 mg of FeSO_4_, 57 mg of ZnSO_4_, 43 mg of MnSO_4_, 23 mg of CuSO_4_, 0.5 mg of CaI, 0.2 mg of CoSO_4_, 0.2 mg of Se, 3,000,000 IU of vitamin A, 330,000 IU of vitamin D_3_, 33,000 IU of vitamin E.

2) *Saccharomyces cerevisiae* and *Lactobacillus plantarum*.

3) Calculated from the chemical composition analysis of individual feed ingredients. The UIP and TDN values were obtained from the mean values presented in the Korean Feeding Standard Establishment Council [2]. UIP and DIP contents of soybean meal and high-protein DDG were determined in our *in situ* experiment [10].

**Table 3:** Treatment effects on performance of Hanwoo steers by production phase

Item	Treatment1)			SE	p-value

	LP	HPLU	HPHU		
Growing phase (8.2–14.3 mo)					
Initial BW (kg)	253	254	254	9.30	0.98
Final BW (kg)	392	404	408	14.7	0.25
Total weight gain (kg)	139	150	154	8.95	0.25
Average daily gain (kg)	0.75	0.81	0.83	0.05	0.14
Daily DM intake (kg)	7.60	8.10	8.40	0.43	0.86
Daily crude protein intake (kg)	0.92^a^	1.22^b^	1.26^b^	0.06	<0.01
Daily DIP intake (kg)	0.60^c^	0.81^a^	0.68^b^	0.03	<0.01
Daily UIP intake (kg)	0.32^c^	0.41^b^	0.58^a^	0.03	<0.01
Feed conversion ratio2) (kg/kg)					
DM	9.40	9.30	9.40	0.45	0.14
Crude protein	1.14^a^	1.40^b^	1.40^b^	0.08	<0.01
Fattening phase (14.3–23.5 mo)					
Final BW (kg)	598	594	604	21.9	0.60
Total weight gain (kg)	206	190	196	9.74	0.60
Average daily gain (kg)	0.73	0.68	0.70	0.03	0.08
Daily DM intake (kg)	9.80	9.70	9.80	0.17	0.90
Daily crude protein intake (kg)	1.16^b^	1.33^a^	1.34^a^	0.02	<0.01
Daily DIP intake (kg)	0.73^b^	0.86^a^	0.74^b^	0.01	<0.01
Daily UIP intake (kg)	0.43^c^	0.47^b^	0.60^a^	0.01	<0.01
Feed conversion ratio (kg/kg)					
DM	11.9	13.1	12.5	0.72	0.13
Crude protein	1.60^b^	2.03^a^	1.93^a^	0.10	<0.01
Finishing phase (23.5–29.3 mo)					
Final BW (kg)	744	739	744	25.1	0.90
Total weight gain (kg)	146	145	140	7.30	0.69
Average daily gain (kg)	0.83	0.83	0.80	0.04	0.66
Daily DM intake (kg)	9.3	8.9	9.1	0.21	0.80
Daily crude protein intake (kg)	1.12	1.17	1.18	0.03	0.53
Daily DIP intake (kg)	0.57^b^	0.62^a^	0.58^b^	0.01	<0.01
Daily UIP intake (kg)	0.55^b^	0.55^b^	0.60^a^	0.01	<0.01
Feed conversion ratio (kg/kg)					
DM	11.7	11.3	12.1	0.63	0.60
Crude protein	1.41^b^	1.48^a^	1.57^ab^	0.07	0.03
Whole period (8.2–29.3 mo)					
Total weight gain (kg)	491	485	490	19.4	0.79
Average daily gain (kg)	0.77	0.76	0.76	0.03	0.44
Daily DM intake (kg)	9.0	8.9	9.0	0.12	0.79
Daily crude protein intake (kg)	1.07^b^	1.24^a^	1.26^a^	0.02	<0.01
Daily DIP intake (kg)	0.64^c^	0.76^a^	0.67^b^	0.01	<0.01
Daily UIP intake (kg)	0.43^c^	0.48^b^	0.59^a^	0.01	<0.01
Feed conversion ratio (kg/kg)					
DM	11.5	11.7	11.8	0.48	0.12
Crude protein	1.38^b^	1.64^a^	1.64^a^	0.06	<0.01

LP, low-protein diet; HPLU, high-protein, low UIP diet; HPHU, high-protein, high UIP diet; SE, standard error; BW, body weight; DM, dry matter; DIP, degraded intake protein; UIP, undegraded intake protein.

1) Analysis of chemical composition of each dietary treatment by production phase is specified in Table 2.

2) Feed conversion ratio was calculated as kg daily nutrient intake divided by average daily gain (kg).

a–c Within a row, values with different letters differ (p<0.05).

**Table 4:** Treatment effects on blood metabolites of Hanwoo steers by production phase

Parameters by cattle age	Treatment1)			SE	p-value

	LP	HPLU	HPHU		
Triglyceride (mg/dL)					
13.6 mo	24.5	24.8	23.9	2.93	0.95
22.5 mo	29.7	30.0	27.8	2.66	0.66
27.5 mo	28.1	29.3	26.1	2.90	0.59
Cholesterol (mg/dL)					
13.6 mo	196	193	184	14.9	0.72
22.5 mo	248^a^	195^b^	223^b^	17.3	0.02
27.5 mo	187^a^	151^b^	169^ab^	10.5	0.03
High-density lipoprotein (mg/dL)					
13.6 mo	>120	>120	>120	-	-
22.5 mo	>120	>120	>120	-	-
27.5 mo	>120	111	116	3.63	0.14
Low-density lipoprotein (mg/dL)					
13.6 mo	36.9	38.0	34.1	5.21	0.74
22.5 mo	62.5^a^	40.9^b^	57.6^a^	7.37	0.02
27.5 mo	45.1^a^	33.5^b^	40.6^a^	3.53	0.03
Glucose (mg/dL)					
13.6 mo	79.4	74.5	74.0	2.92	0.14
22.5 mo	73.8	73.7	74.6	2.11	0.89
27.5 mo	60.9	51.1	48.5	4.23	0.06
Total protein (g/dL)					
13.6 mo	6.8	6.7	6.8	0.15	0.82
22.5 mo	7.0	6.8	6.8	0.13	0.13
27.5 mo	6.9	6.8	7.0	0.17	0.53
Albumin (g/dL)					
13.6 mo	4.1	4.0	4.1	0.06	0.16
22.5 mo	4.0	3.8	3.9	0.08	0.32
27.5 mo	4.0	4.0	4.1	0.07	0.48
Globulin (g/dL)					
13.6 mo	2.8	2.7	2.7	0.12	0.59
22.5 mo	3.1	3.0	2.9	0.12	0.34
27.5 mo	2.9	2.8	2.9	0.14	0.78
Urea-N (mg/dL)					
13.6 mo	18.4	15.2	16.8	1.25	0.06
22.5 mo	17.4^ab^	18.7^a^	16.2^b^	0.92	0.03
27.5 mo	17.2	16.4	17.4	1.07	0.65
Creatinine (mg/dL)					
13.6 mo	1.2	1.1	1.1	0.05	0.29
22.5 mo	1.2	1.1	1.2	0.05	0.46
27.5 mo	1.3	1.3	1.3	0.07	0.71
Urea-N:creatinine					
13.6 mo	15.8	13.9	15.3	1.40	0.38
22.5 mo	14.9^ab^	17.0^a^	14.3^b^	0.95	0.02
27.5 mo	13.1	12.8	14.0	1.12	0.59

LP, low-protein diet; HPLU, high-protein, low UIP diet; HPHU, high-protein, high UIP diet; SE, standard error.

1) Analysis of chemical composition of each dietary treatment by production phase is specified in Table 2.

a,b Within a row, values with different letters differ (p<0.05).

**Table 5:** Treatment effects on serum enzymes, hematological parameters, and electrolyte composition of Hanwoo steers by production phase

Parameters by cattle age	Treatment1)			SE	p-value

	LP	HPLU	HPHU		
Alkaline phosphatase (U/L)					
13.6 mo	154	156	159	19.9	0.97
22.5 mo	132	128	129	13.4	0.95
27.5 mo	96.7	102	108	10.8	0.69
Lactate dehydrogenase (U/L)					
13.6 mo	>1,200	>1,200	>1,200	-	-
22.5 mo	>1,200	>1,200	>1,200	-	-
27.5 mo	>1,200	>1,200	1,185	13.2	0.48
White blood cell count (10^6^/mL)					
13.6 mo	9.0	10.1	11.4	1.07	0.14
22.5 mo	10.6	10.3	9.3	0.92	0.35
27.5 mo	8.5	9.5	9.2	0.82	0.60
Red blood cell count (10^6^/μL)					
13.6 mo	8.0	9.0	9.1	0.84	0.41
22.5 mo	9.1	8.5	8.0	0.49	0.10
27.5 mo	8.8	8.7	8.6	0.39	0.91
Platelet count (10^6^/mL)					
13.6 mo	248	250	290	34.0	0.47
22.5 mo	247	267	240	28.6	0.62
27.5 mo	223	207	227	40.7	0.98
Calcium (Ca^+^) (mg/dL)					
13.6 mo	10.9	10.6	10.8	0.13	0.08
22.5 mo	10.3	10.2	10.1	0.14	0.32
27.5 mo	10.3	10.4	10.6	0.29	0.70
Inorganic phosphorus (P^−^) (mg/dL)					
13.6 mo	8.2	8.6	8.2	0.35	0.43
22.5 mo	7.2	7.1	7.4	0.20	0.29
27.5 mo	7.3	7.8	8.2	0.29	0.07
Potassium (K^+^) (mmol/L)					
13.6 mo	5.3	5.2	5.2	0.17	0.68
22.5 mo	4.9	4.9	5.0	0.14	0.29
27.5 mo	5.1	5.2	5.5	1.99	0.22
Sodium (Na^+^) (mmol/L)					
13.6 mo	142	142	142	0.68	0.70
22.5 mo	141	143	142	1.28	0.45
27.5 mo	146	146	146	0.99	0.90
Chlorine (Cl^−^) (mmol/L)					
13.6 mo	99.2	98.9	99.2	0.45	0.82
22.5 mo	97.0	98.5	98.5	1.04	0.27
27.5 mo	101	99.4	99.9	0.73	0.16

LP, low-protein diet; HPLU, high-protein, low UIP diet; HPHU, high-protein, high UIP diet; SE, standard error.

1) Analysis of chemical composition of each dietary treatment by production phase is specified in Table 2.

**Table 6:** Treatment effects on carcass quality parameters of Hanwoo steers evaluated by real-time ultrasound at an age of 24 mo

Items	Treatment1)			SE	p-value

	LP	HPLU	HPHU		
Backfat thickness (mm)	10.1	8.2	9.6	1.29	0.30
Longissimus muscle area (cm^2^)	85.8	89.6	89.6	2.22	0.16
Yield grade2)	1.7	1.2	1.5	0.10	0.08
Marbling score3)	11.3	11.1	14.0	1.38	0.08
Quality grade4)	3.2	3.2	2.8	0.24	0.14
1^++^, head (%)	-	-	-	-	-
1^+^, head (%)	1 (8.3)	2 (16.7)	3 (25.0)	-	-
1, head (%)	8 (66.7)	6 (50.0)	9 (75.0)	-	-
2, head (%)	3 (25.0)	4 (33.3)	-	-	-

LP = low-protein diet; HPLU = high-protein, low UIP diet; HPHU = high-protein, high UIP diet; SE, standard error.

1) Analysis of chemical composition of each dietary treatment by production phase is specified in Table 2.

2) Converted into numeric values: grade A = 1, B = 2, and C = 3.

3) 1 = trace, 27 = abundant.

4) Grade 1^++^ represents the highest quality, whereas grade 2 represents the lowest quality.

**Table 7:** Treatment effects on carcass characteristics of Hanwoo steers slaughtered at an age of 29.3 mo

Items	Treatment1)			SE	p-value

	LP	HPLU	HPHU		
Cold carcass weight (kg)	420	408	420	16.8	0.72
Yield traits					
Backfat thickness (mm)	13.6	13.2	13.8	1.31	0.88
Longissimus muscle area (cm^2^)	92.1	98.5	97.8	4.47	0.31
Yield index2)	64.8	64.4	65.4	1.63	0.81
Yield grade3)	2.1	1.7	1.8	0.24	0.21
Quality traits					
Marbling score4)	5.8^ab^	5.0^b^	6.8^a^	0.72	0.05
Meat color5)	4.8	4.9	4.8	0.19	0.67
Fat color score6)	3.0	3.0	3.0	-	-
Texture score7)	1.2	1.3	1.0	0.15	0.09
Maturity score8)	2.1	2.3	2.3	0.17	0.52
Quality grade9)	2.4	2.8	1.9	0.35	0.07
1^++^, head (%)	2 (16.7)	1 (8.33)	3 (25.0)	-	-
1^+^, head (%)	4 (33.3)	4 (33.3)	7 (58.3)	-	-
1, head (%)	5 (41.7)	4 (33.3)	2 (16.7)	-	-
2, head (%)	1 (8.33)	3 (25.0)	-	-	-

LP = low-protein diet; HPLU = high-protein, low UIP diet; HPHU = high-protein, high UIP diet; SE, standard error.

1) Analysis of chemical composition of each dietary treatment by production phase is specified in Table 2.

2) Calculated as = 68.184 − (0.625 × back fat thickness, mm) + (0.130 × *longissimus* muscle area, cm^2^) − (0.024 × cold carcass weight, kg) + 3.23.

3) Converted into numeric values as: grade A = 3, B = 2, and C = 1. A (high yield; >67.5), B (yields <67.5 and >62.0), and C (low yield; <67.5).

4) 1 = trace, 9 = abundant.

5) 1 = bright cheery red, 7 = extremely dark red.

6) 1 = white, 7 = yellow.

7) 1 = soft, 3 = firm.

8) 1 = immature, 9 = mature.

9) Converted to numeric values: grade 1^++^ = 1, 1^+^ = 2, 1 = 3, and 2 = 4. Grade 1^++^ represents the highest quality, whereas grade 2 represents the lowest quality.

a,b Within a row, values with different letters differ (p<0.05).

**Table 8:** Average feed cost and calculations of production costs and net income of Hanwoo steers receiving the different diets

Items	Treatment1)			SE	p-value

	LP	HPLU	HPHU		
Average feed cost ($/d/head)2)					
Growing (Ages 8.2–14.3 mo)	3.24	3.51	3.42	0.18	0.39
Fattening (Ages 14.3–23.5 mo)	3.68	3.88	3.81	0.07	0.08
Finishing (Ages 23.5–29.3 mo)	3.39	3.32	3.33	0.07	0.63
Whole (Ages 8.2–29.3 mo)	3.44	3.57	3.52	0.06	0.16
Economic calculations					
Gross receipts3) ($)	6,715	6,480	6,973	-	-
Index (%)	100.0	96.5	103.8	-	-
Operating costs ($)	4,068	4,115	4,129	-	-
Index (%)	100.0	101.2	101.5	-	-
Livestock4) ($)	1,849	1,808	1,854	-	-
Feed ($)	2,218	2,308	2,275	-	-
Net income5) ($)	2,646	2,365	2,844	-	-
Index (%)	100.0	89.4	107.5	-	-

LP = low-protein diet; HPLU = high-protein, low UIP diet; HPHU = high-protein, high UIP diet; SE, standard error.

1) Analysis of chemical composition of each dietary treatment by production phase is specified in Table 2.

2) Exchange rate: ₩1,211.5 = 1 USD.

3) Selling price of carcass by grade and the associated by-products.

4) Average purchased price of animals in the livestock market.

5) Net income from sale of finishing steer, calculated as the difference between gross receipts and operating costs.
